# Maresin 1 Attenuates Lipopolysaccharide-Induced Acute Kidney Injury via Inhibiting NOX4/ROS/NF-κB Pathway

**DOI:** 10.3389/fphar.2021.782660

**Published:** 2021-12-10

**Authors:** Jiameng Li, Zhuyun Zhang, Liya Wang, Luojia Jiang, Zheng Qin, Yuliang Zhao, Baihai Su

**Affiliations:** Department of Nephrology, West China Hospital, Sichuan University, Chengdu, China

**Keywords:** acute kidney injury, lipopolysaccharide, maresin 1, inflammation, apoptosis, mitochondrial dysfunction

## Abstract

Sepsis-associated acute kidney injury (S-AKI) is a common complication in hospitalized and critically ill patients, which increases the risk of multiple comorbidities and is associated with extremely high mortality. Maresin 1 (MaR1), a lipid mediator derived from the omega-3 fatty acid docosahexaenoic acid has been reported to protect against inflammation and promote the regression of acute inflammation. This study proposed to systematically investigate the renoprotective effects and potential molecular mechanism of MaR1 in septic acute kidney injury. We established a S-AKI animal model by a single intraperitoneal injection of lipopolysaccharide (LPS), 10 mg/kg, on male C57BL/6J mice. LPS-stimulated (100 μg/ml) mouse kidney tubular epithelium cells (TCMK-1) were used to simulate septic AKI *in vitro*. The results showed that pretreatment with MaR1 significantly reduced serum creatinine and blood urea nitrogen levels as well as tubular damage scores and injury marker neutrophil gelatinase-associated lipocalin in septic AKI mice. Meanwhile, MaR1 administration obviously diminished pro-inflammatory cytokines (TNF-α, IL-6, IL-1β, and MCP-1), downregulated BAX and cleaved caspase-3 expression, and upregulated BCL-2 expression in the injured kidney tissues and TCMK-1 cells. In addition, MaR1 reduced malondialdehyde production and improved the superoxide dismutase activity of renal tissues while inhibiting reactive oxygen species (ROS) production and protecting the mitochondria. Mechanistically, LPS stimulated the expression of the NOX4/ROS/NF-κB p65 signaling pathway in S-AKI kidneys, while MaR1 effectively suppressed the activation of the corresponding pathway. In conclusion, MaR1 attenuated kidney inflammation, apoptosis, oxidative stress, and mitochondrial dysfunction to protect against LPS-induced septic AKI via inhibiting the NOX4/ROS/NF-κB p65 signaling pathway.

## Introduction

Acute kidney injury (AKI) is a critical clinical condition affecting multiple organs and systems, which is characterized by the abrupt deterioration in renal function due to various reasons. Sepsis, as a life-threatening organ dysfunction, is the leading cause of AKI in hospitalized and critically ill patients. It is found that sepsis accounts for more than 50% of patients with AKI (Hoste et al., 2006; Uchino et al., 2006). Moreover, sepsis-associated acute kidney injury (S-AKI) increases the risk of developing chronic comorbidities and is related to extremely high mortality (Uchino et al., 2005; Bagshaw et al., 2007; Bouchard et al., 2015; Hoste et al., 2015). Despite this, unfortunately, the pathophysiologic mechanisms of S-AKI have not been fully understood. The treatment remains reactive and nonspecific, and no available preventive therapies exist (Peerapornratana et al., 2019). Therefore, it is very urgent to search for new effective therapeutic options for S-AKI.

In recent years, following the discovery of arachidonic acid-derived lipoxins, resolvins, and protectins, a new family of specialized pro-resolving lipid mediators (SPMs) called maresins has been discovered in the inflammatory exudates and lipid mediators during the subsiding stage (Serhan et al., 2009). Many studies have shown the benefits of these SPMs that can limit the tissue infiltration of polymorphonuclear leukocytes (PMNs), reduce collateral tissue damage by phagocytes, shorten the resolution interval, enhance macrophage phagocytosis and efferocytosis, and counter-regulate proinflammatory chemical mediators (Serhan, 2017). Maresins (MaRs) have already been proved to play a role of initiating the resolution programs of acute inflammation, thereby reducing granulocyte trafficking and the production of cytokines and extracellular reactive oxygen species (ROS) as well as decreasing the magnitude of the overall inflammatory response by enhancing the macrophage-mediated clearance of cellular debris and invading microbes (Serhan, 2014). Maresin 1 (MaR1), which was the first maresin to be identified, is biochemically synthesized from 14-lipoxygenation of DHA by human macrophage 12-lipoxygenase (12-LOX) that produces14-hydroperoxy-docosahexaenoic acid (Serhan et al., 2012). During septic shock, many inflammatory mediators, such as tumor necrosis factor-α (TNF-α) and interleukin (IL)-1β as well as ROS, increase drastically and lead to tissue or organ damages (Seely et al., 2011; Wang et al., 2012; Peerapornratana et al., 2019). Recent investigations have shown that MaR1 can promote the resolution of acute inflammation and oxidative stress in sepsis (Gong et al., 2015; Li et al., 2016; Gu et al., 2018; Sun et al., 2019). Nevertheless, the renoprotective effects and specific mechanisms of MaR1 in S-AKI still remain uncertain.

NADPH oxidase 4 (NOX4) is a constitutive enzyme specialized in controlling oxidative stress response (Cornelius et al., 2014). Emerging evidence reveals that the upregulation of NOX4 is primarily responsible for the increase of ROS and plays a critical role in mediating mitochondrial dysfunction and cell apoptosis (Kuroda et al., 2010). ROS produced by the mitochondria and mitochondrial dysfunction have been proposed in the pathogenesis of sepsis. NOX4 is a major renal isoform and highly expressed in the kidney (Geiszt et al., 2000). Studies have shown that ROS derived from NADPH oxidase contribute to a wide variety of renal diseases, such as diabetic nephropathy, acute kidney injury, and obstructive nephropathy (Kleikers et al., 2012; Swärd and Rippe, 2012; Xu et al., 2013; Wang et al., 2020). In addition, ROS could modulate signaling molecules and transcription factors (Filomeni et al., 2005; Sadoshima, 2006). The NF-κB signaling pathway is a downstream pathway of ROS, which can regulate inflammation, oxidative stress, and apoptosis (Kong et al., 2019; Zhu et al., 2021). Chatterjee et al. (2014) discovered that MaR1 could reduce NOX expression and ROS level as well as inhibit NF-κB activation in human vascular smooth muscle cells and endothelial cells. Therefore, we speculated that MaR1 may alleviate renal injury in S-AKI *via* the NOX4/ROS/NF-κB signaling pathway.

Lipopolysaccharide (LPS), an endotoxin derived from the outer membrane of Gram-negative bacteria, is widely used in the establishment of sepsis and a S-AKI animal model. In the current study, we established a LPS-induced S-AKI mouse model and use LPS-stimulated TCMK-1 cells to investigate the renoprotective effects of MaR1 and its related molecular mechanism so as to provide potential insights of therapeutic targets to S-AKI.

## Materials and Methods

### Reagents and Antibodies

LPS (L2630, *E. coli* 0111: B4) was purchased from Sigma-Aldrich (St. Louis, MO, United States). MaR1 was obtained from Cayman Chemical (Ann Arbor, MI, United States). The primary antibodies used in this study were as follows: anti-NOX4 (ab133303, Abcam), anti-TNF-α (AF7014; Affinity), anti-IL-6 (347023; Zenbio), anti-MCP-1 (AF-479-SP, R&D), anti-BAX (50599-2-Ig; Proteintech), anti-BCL-2 (26593-1-AP; Proteintech), anti-cleaved caspase-3 (9661; Cell Signaling Technology), anti-DRP-1 (NB110-55288; Novus), anti-MFN-1 (13798-1-AP; Proteintech), anti-OPA-1 (NB110-55290; Novus), anti-p-IκBα (AF2002; Affinity), anti-IκBα (AF5002; Affinity), anti-p-NF-κB P65 (3033; Cell Signaling Technology), anti-NF-κB P65 (sc-8008; Santa Cruz), and anti-GAPDH (ab8245, Abcam).

### Animal Experiments

Male C57BL/6J mice (6–8 weeks, weight: 20–25 g) were purchased from Chengdu Dossy Experimental Animals (Chengdu, China). The mice were housed at the Animal Experiment Center of West China Hospital, Sichuan University (Chengdu, China). S-AKI was induced by LPS treatment. The mice were intraperitoneally injected with LPS (10 mg/kg), and the control mice were injected with 0.9% saline (Wang et al., 2021). As for the MaR1 group, the mice were pretreated i.p. with MaR1 (5 μg/kg) 30 min prior to LPS administration. The blood and kidneys were harvested at 6, 12, 24, and 48 h, respectively, with the mice anesthetized by pentobarbital sodium injection and then sacrificed humanely. The levels of serum creatinine (Scr) and blood urea nitrogen (BUN) were examined by automatic biochemical analyzer (Chemray 240, Rayto Life and Analytical Sciences, Shenzhen, China). The AKI model was successfully established when Scr rose up to twofold of their control littermates.

### Cell Culture and Treatments of TCMK-1

Mouse kidney tubular epithelium cells (TCMK-1, ATCC^®^ CCL-139™) were purchased from American Type Culture Collection (Manassas, VA, United States) and cultured in Minimum Essential Medium (MEM) (Gibco, Rockville, MD, United States) supplemented with 10% fetal bovine serum (FBS) at 37°C under an atmosphere of 5% CO_2_. For LPS treatment, TCMK-1 cells at a confluence of 50–60% were incubated in MEM with 0.5% FBS for 24 h. Then, the cells were exposed to LPS (100 μg/ml) for another 24 h. In the MaR1 group, the cells were pretreated with MaR1 (100 nM) 30 min prior to LPS administration.

### Cell Viability Assay

Cell viability was identified in this experiment relying on CCK-8 assay (Dojindo Molecular Technologies, Gaithersburg, MD). According to the instruction, TCMK-1 cells (5,000 cells/well) were seeded into 96-well plates for 24 h in an environment containing 5% CO_2_ at 37°C and later incubated with LPS and MaR1 at various concentrations for another 24 h. Then, the culture medium in each well was replaced with medium containing 10 μl CCK-8 solutions and incubated without light for 1 h under the same conditions. After that, the absorbance of the solution in each well was detected at 450 nm wave length by a microplate reader (Synergy Mx, Biotek, Winooski, VT, United States).

### Reactive Oxygen Species Detection

The ROS in kidney tissue was detected by fluorescence microscope (Nikon, Tokyo, Japan) with the oxidative fluorescent dye dihydroethidium (Sigma-Aldrich, St. Louis, MO, United States) staining *in situ*. The nuclei were counterstained with 4,6-diamidino-2-phenylindole (DAPI) (Servicebio, Wuhan, China). The ROS in TCMK-1 cells was measured by flow cytometry (Beckman Coulter, Brea, CA) using 2′,7′-dichlorofluorescein diacetate according to the manufacturer’s guidelines (Beyotime Biotechnology, Shanghai, China).

### Mitochondrial Complex I Activity Assay

The activity of mitochondrial complex I was determined by the Micro Mitochondrial Respiratory Chain Complex I Activity Assay Kit (Solarbio Technology, Beijing, China). Briefly, renal tissue homogenates were added into the reaction buffer, respectively. The reaction mixture was quickly mixed and transferred to a prewarmed (37°C) quartz cuvette and immediately put into a spectrophotometer. The absorbance of the reaction mixture was measured at 340 nm. We recorded the absorbance value A1 at the 10th second, then accurately reacted in the environment of 37°C for 2 min, quickly took it out, and recorded the absorbance value A2 at 2 min. A bicinchoninic acid (BCA) protein assay kit (Biosharp, Hefei, China) was utilized to measure the protein concentration of the renal tissue homogenates. Finally, we calculated the mitochondrial complex I activities according to the provided formula.

### ATP Production Assay

The ATP production level of kidney tissue was detected by ATP Assay Kit (Beyotime Biotechnology, Shanghai, China) according to the instructions. Kidney tissues were lysed by a glass homogenizer on ice and then centrifuged at 12,000 *g* for 5 min. The supernatant was collected for luminescence by a multifunctional enzyme marking instrument. In order to eliminate the error caused by the difference of protein content in the sample preparation, a BCA protein assay kit (Biosharp, Hefei, China) was utilized to measure the concentration of proteins in the samples. Finally, we converted the concentration of ATP into millimole per milligram of protein.

### ELISA

The TNF-α, IL-6, and IL-1β levels in the serum of mice or TCMK-1 cell supernatant and the malondialdehyde (MDA) and superoxide dismutase (SOD) levels of renal tissue homogenates were detected by Mouse TNF-α ELISA kit (Neobioscience technology, Shenzhen, China), Mouse IL-6 Uncoated ELISA (Thermo Fisher Scientific, Vienna, Austria), Mouse IL-1 beta Uncoated ELISA (Thermo Fisher Scientific, Vienna, Austria), MDA assay kit (Nanjing Jiancheng Bioengineering Institute, Nanjing, China), and total superoxide dismutase assay kit (Nanjing Jiancheng Bioengineering Institute, Nanjing, China) according to standard protocol of the manufacturer.

### Quantitative Real-Time Polymerase Chain Reaction Analysis

Total RNA was extracted from frozen kidney tissues or TCMK-1 cells using the total RNA isolation kit (Vazyme, Nanjing, China) according to the instructions of the manufacturer. Complementary DNA was synthesized by using HiScript II Q Select RT SuperMix for qPCR (Vazyme, Nanjing, China). For the purposes of qRT-PCR analysis, GAPDH was used as the reference. The mRNA levels of neutrophil gelatinase-associated lipocalin (NGAL), TNF-α, IL-6, MCP-1, BAX, BCL-2, DRP-1, and OPA-1 were analyzed using qRT-PCR and the SYBR Green Supermix (Vazyme, Nanjing, China). The primers were synthesized by Tsingke Biotechnology, and the sequences are listed in Table 1. All treatments and conditions were performed in triplicate to calculate the statistical significance, and the results were calculated using the 2^−ΔΔCt^ method.

**TABLE 1:** Sequences of the primers for quantitative real-time PCR.

Mouse gene	Sequence
*F-NGAL*	GGA​ACG​TTT​CAC​CCG​CTT​TG
*R-NGAL*	CCA​CAC​TCA​CCA​CCC​ATT​CA
*F-TNF-α*	ACC​CTC​ACA​CTC​AGA​TCA​TCT​TC
*R-TNF-α*	TGG​TGG​TTT​GCT​ACG​ACG​T
*F-IL-6*	ACA​ACC​ACG​GCC​TTC​CCT​ACT​T
*R-IL-6*	CAC​GAT​TTC​CCA​GAG​AAC​ATG​TG
*F-MCP-1*	TTA​AAA​ACC​TGG​ATC​GGA​ACC​AA
*R-MCP-1*	GCA​TTA​GCT​TCA​GAT​TTA​CGG​GT
*F-BAX*	TGA​AGA​CAG​GGG​CCT​TTT​TG
*R-BAX*	AAT​TCG​CCG​GAG​ACA​CTC​G
*F-BCL-2*	GTC​GCT​ACC​GTC​GTG​ACT​TC
*R-BCL-2*	CAG​ACA​TGC​ACC​TAC​CCA​GC
*F-DRP-1*	AGA​TGA​CCA​CCA​CTG​TAG​CC
*R-DRP-1*	AGC​TTC​CCC​TTT​CCC​TGT​TT
*F-OPA-1*	CTT​CGT​CTC​TCC​TCA​TCG​GG
*R-OPA-1*	TGA​CAT​CCC​ACG​CTG​TAC​AG
*F-GAPDH*	AGGTCGGTGAACGGATTG
*R-GAPDH*	TGT​AGA​CCA​TGT​AGT​TGA​GGT​CA

### Western Blot Assay

Total proteins of kidney tissues or TCMK-1 cells were extracted using radio-immune precipitation assay lysis buffer (Beyotime Biotechnology, Shanghai, China) supplemented with protease inhibitor cocktail and phosphatase inhibitor cocktail (Bimake, Houston, TX, United States). The protein concentrations were determined using the BCA protein assay kit (Biosharp, Hefei, China). Equal amounts of protein lysate were separated by 10–12% SDS-polyacrylamide gel in Tris/SDS buffer and then transferred onto polyvinylidene difluoride membranes (Millipore, Billerica, MA, United States). The membranes were blocked in 5% non-fat milk (w/v) in Tris-buffered saline with 0.1% Tween-20 (TBS-T) for 1 h at room temperature and incubated with corresponding primary antibodies at 4°C overnight. After washing, the membranes were further incubated with HRP-conjugated secondary antibodies (1:10,000) at room temperature for 1 h. The immunoreactive bands were evaluated to visualize the expression of designated proteins using the chemiluminescence detection system through the peroxidase reaction, and the images of the bands were recorded with the Chemi Doc MP imaging system (Bio-Rad, United States). GAPDH was used as the internal loading control. The films were analyzed with ImageJ software (National Institute of Health, Bethesda, MD, United States). All experiments were repeated at least three times.

### Renal Histology Staining and Evaluation

Kidney tissues were fixed with 4% paraformaldehyde for paraffin embedding, and kidney sections of 4 μm were used for hematoxylin–eosin (HE) staining. HE-stained tissue sections were viewed by light microscopy at magnifications of ×200 or ×400. Tissue damage was scored in a blinded manner by the percentage of injured renal tubules and histological injury that was indicated by brush border lost, tubular dilation/flattening, tubular degeneration, tubular cast formation, and vacuolization. Tissue injury was scored on a scale of 0–4, with 0, 1, 2, 3, and 4 corresponding to 0, <25, 26–50, 51–75, and >76% of injured/damaged renal tubules, respectively. Ten fields of ×400 magnification were examined and averaged.

### Immunohistochemistry Assay

Tissue sections were dewaxed, dehydrated, and washed with phosphate-buffered saline (PBS). After removing endogenous peroxidase with 3% H_2_O_2_, citrate was used for antigen retrieval. The primary antibody of anti-NOX4 (1:100) was incubated overnight at 4°C. After washing three times with PBS, a biotinylated secondary antibody was used to incubate the slices at room temperature for 30 min. Images of the random renal cortex sections were observed and captured with an AxioCamHRc digital camera (Carl Zeiss, Jena, Germany).

### Immunofluorescence Staining

Renal specimens were embedded in OCT compound and cut into 4-μm sections on a cryostat and stored at −80°C until use. Non-specific binding sites were blocked with PBS containing 5% bovine serum for 1 h at room temperature. Then, the sections were labeled with the indicated primary antibodies in a humidified chamber overnight at 4°C. After washing with PBS, the corresponding secondary antibodies were applied for 1 h. The nuclei were counterstained with DAPI (Servicebio, Wuhan, China). Images were exported from ZEN 2012 microscopy software (blue edition).

### TUNEL Assay

Kidney cortex cell and cultured TCMK-1 cell apoptosis were examined by TUNEL assay using the TUNEL kit according to the instructions of the manufacturer (Roche, Basel, Switzerland). The nuclei were counterstained with DAPI. Positive staining was detected by fluorescence microscopy (Carl Zeiss, Jena, Germany). Ten randomly selected fields were counted to determine the number of apoptotic nuclei.

### Cell Apoptosis Assay

The apoptotic rate of cultured TCMK-1 cells was measured by Annexin V-FITC/PI kit (4A Biotech, Beijing, China) combined with flow cytometry (Beckman Coulter, Brea, CA, USA). Cell preparation was conducted according to standard instructions from the manufacturer. Briefly, the cells were washed twice with cold PBS and then resuspended in 100 μl 1× binding buffer. Then, 5 μl of Annexin V was added to the cell suspension. The cells were then subjected to gentle vortexing and then incubation for 15 min at room temperature in the dark. Finally, 5 μl PI and 400 μl of 1× binding buffer were added, and the cells were analyzed by flow cytometry within 1 h.

### Mitochondrial Morphology Observation by Transmission Electron Microscopy

In brief, 1 mm^3^ fresh kidney cortex was removed, fixed in cold 2.5% glutaraldehyde for 2 h at 4°C, and then treated according to standard procedures, including dehydration, osmosis, embedding, sectioning, and staining. The ultrastructure of renal cells was observed using a Hitachi H-7650 electron microscope.

### Measurement of Mitochondrial Membrane Potential

The mitochondrial membrane potential (MMP) of TCMK-1 cells was determined with mitochondrial membrane potential assay kit with JC-1 (Beyotime, Shanghai, China) according to the instructions of the manufacturer. Briefly, after the TCMK-1 cells were processed, JC-1 working dye solution was added and mixed well, and the cells were incubated without light at 37°C for 30 min. Then, the cells were washed with cold staining buffer. Afterwards, the MMP of different samples was detected by fluorescence microscopy (Carl Zeiss, Jena, Germany) or flow cytometry (Beckman Coulter, Brea, CA, USA). Mitochondrial depolarization was determined by increases in red/green fluorescence intensity ratios.

### Statistical Analysis

All experiments were repeated three times unless otherwise stated. Data are expressed as means ± standard deviation (SD) of the indicated number of independent experiments. ANOVA, followed by Tukey’s post-tests, was used to determine the statistical differences among multiple groups. Statistical analysis was determined with the GraphPad Prism software (ver. 6.01; GraphPad, San Diego, CA, United States). Data were considered significant when *p* <0.05.

## Results

### MaR1 Protects Renal Function and Ameliorates Pathological Injury in LPS-Induced S-AKI Mice

**FIGURE 1:**
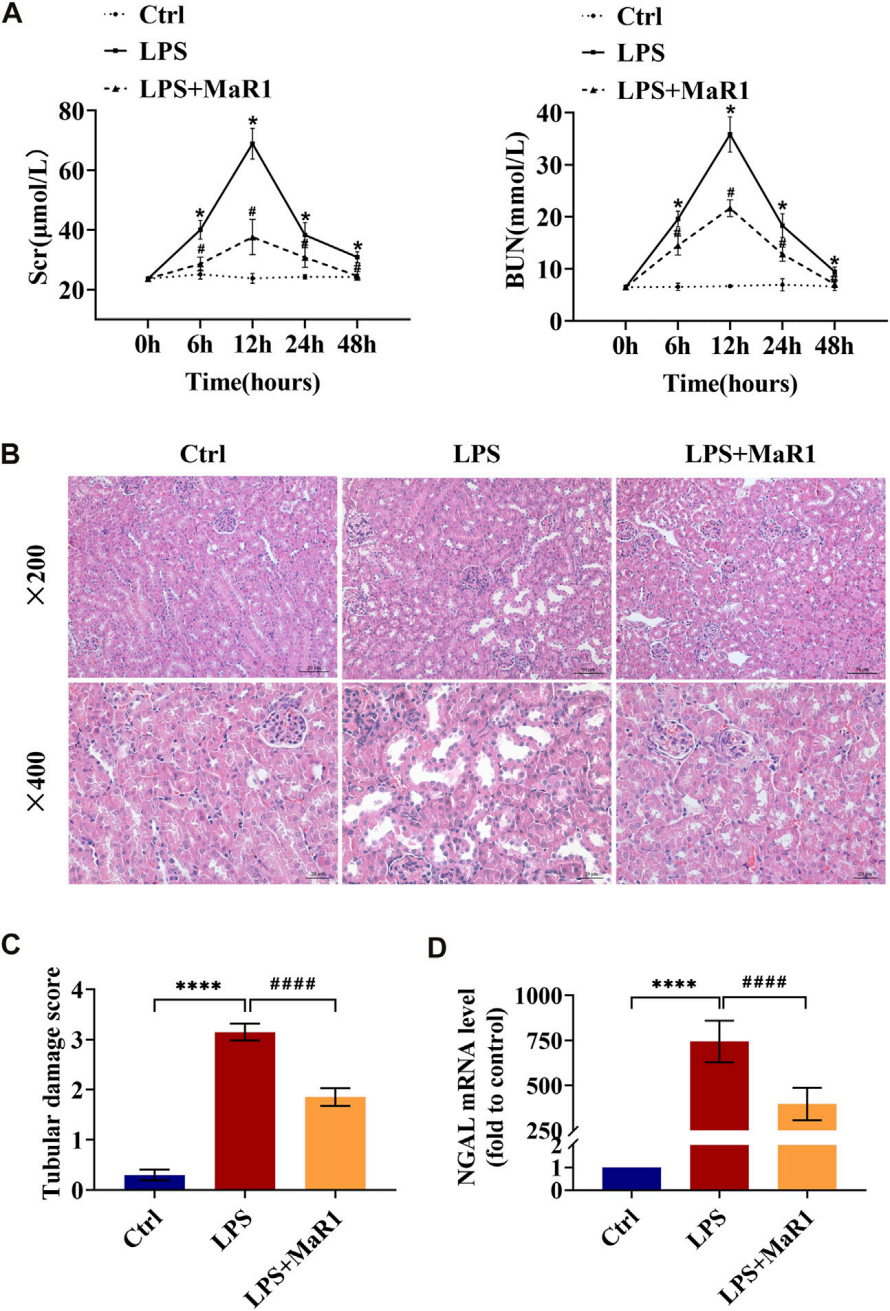
MaR1 alleviated renal injury in lipopolysaccharide (LPS)-induced S-AKI mice. **(A)** Serum creatinine and blood urea nitrogen levels at 0, 6, 12, 24, and 48 h after LPS intraperitoneal injection in each group of mice. **(B)** Representative HE staining images of kidney tissues were collected (scale bars = 50 and 20 μm) (time point: 12 h). **(C)** Pathological tubular damage score. **(D)** Gene expression of neutrophil gelatinase-associated lipocalin in renal tissues were quantitated using qRT-PCR (time point: 12 h). Data are presented as mean ± SD, *n* = 5. *p < 0.05, **p < 0.01, ***p < 0.001, ****p < 0.0001; #*p* < 0.05, ##*p* < 0.01, ###*p* < 0.001, ####*p* < 0.0001 *vs*. LPS group.

To determine the protective effects of MaR1, an effective mouse model of LPS-induced AKI was established, and renal function as well as pathological condition was evaluated. As shown in Figure 1A, the Scr and BUN levels of the LPS group became higher than that of the control group from 6 h, came to peak at 12 h, then started to fall at 24 h, and returned to normal at 48 h. Therefore, we selected a 12-h point in the follow-up research. The administration of MaR1 significantly attenuated the increase of Scr and BUN levels in S-AKI mice at 6, 12, and 24 h (*p* < 0.05). Meanwhile, MaR1 effectively alleviated kidney pathological damage characterized by the dilation of tubular lumen, tubular epithelial vacuolation/flatness, loss of brush border, and exposure of epithelial nucleus compared to that of the LPS group by HE staining (Figures 1B,C). NGAL is a novel biomarker of tubular injury reflecting kidney damage, whose mRNA expression was dramatically increased in kidneys after LPS exposure for 12 h. Treatment with MaR1 remarkably reduced the mRNA level of NGAL in kidneys (Figure 1D). These findings illustrated that MaR1 could effectively improve the renal dysfunction and pathological damage in LPS-stimulated septic AKI mice.

### MaR1 Reduces Inflammation in LPS-Induced S-AKI Mice

**FIGURE 2:**
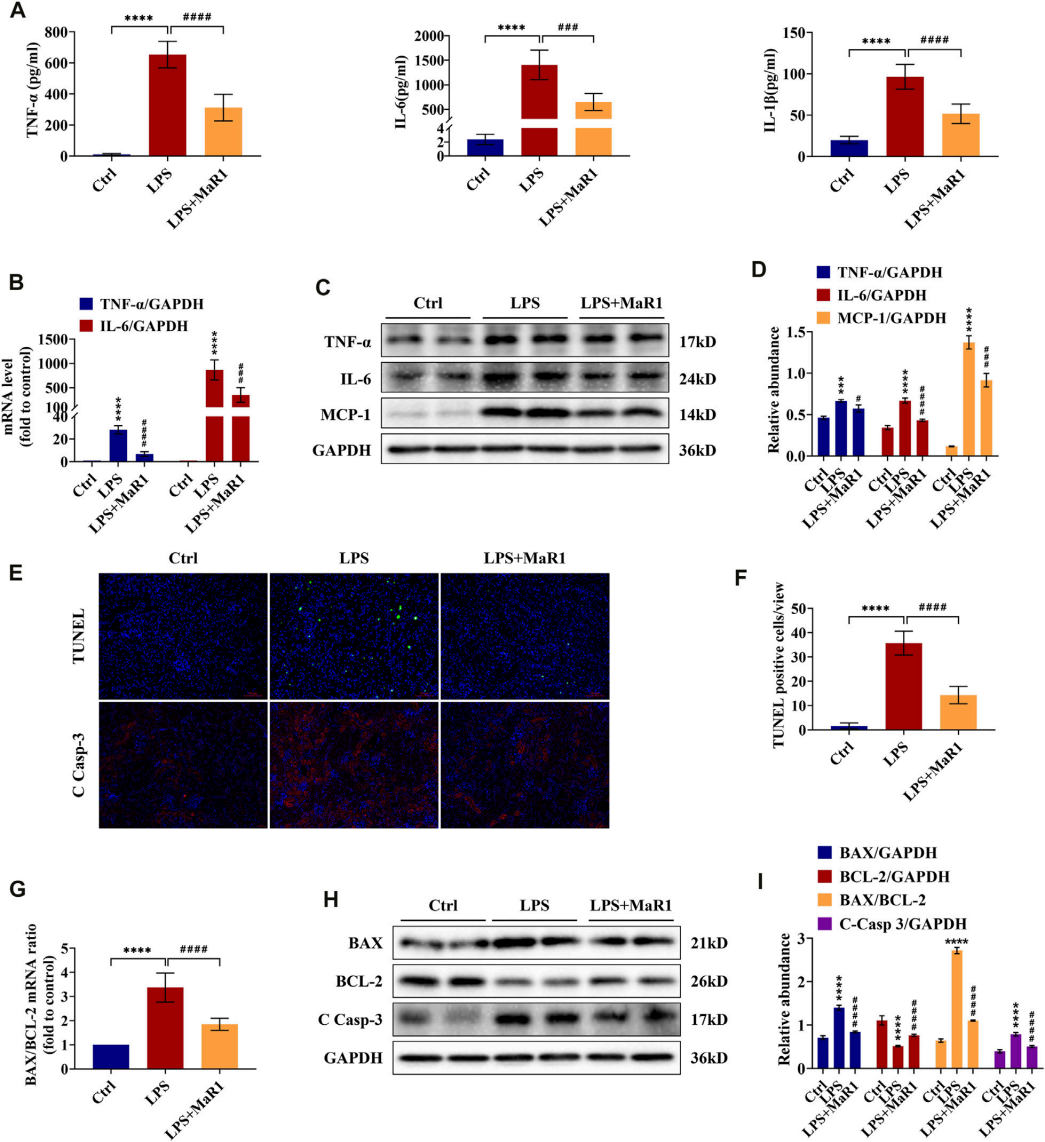
MaR1 inhibited inflammation and renal cell apoptosis in lipopolysaccharide (LPS)-induced S-AKI mice. **(A)** The serum levels of TNF-α, IL-6, and IL-1β were determined using assay kits in each group of mice. **(B)** Gene expression of TNF-α and IL-6 in renal tissues was quantitated using qRT-PCR. **(C)** The expressions of TNF-α, IL-6, and MCP-1 in renal tissues were analyzed using Western blot. **(D)** The protein levels of TNF-α, IL-6, and MCP-1 were quantified by densitometry and normalized with GAPDH. **(E)** TUNEL staining and immunofluorescence staining images of cleaved caspase-3 (C Casp-3) expression in renal sections were collected (scale bars = 50 μm). **(F)** TUNEL staining positive cells were counted in each group. **(G)** The gene expression ratio of BAX/BCL-2 in renal tissues was quantitated using qRT-PCR. **(H)** The expressions of BAX, BCL-2, and C Casp-3 in renal tissues were analyzed using Western blot. **(I)** The protein levels of BAX, BCL-2, and C Casp-3 were quantified by densitometry and normalized with GAPDH (time point: 12 h). Data are presented as mean ± SD, *n* = 5. *p < 0.05, **p < 0.01, ***p < 0.001, ****p < 0.0001 *vs*. control group; #*p* < 0.05, ##*p* < 0.01, ###*p* < 0.001, ####*p* < 0.0001 *vs*. LPS group.

Inflammation at the site of kidney tissue injury is a hallmark of renal injury during sepsis. To evaluate whether MaR1 could reverse the LPS-evoked inflammatory cascade, we employed ELISA, qRT-PCR, and Western blot to detect the production of pro-inflammatory cytokine in the serum and kidneys of S-AKI mice. As shown in Figures 2A–D, the serum levels of TNF-α, IL-6, and IL-1β, the mRNA levels of TNF-α and IL-6, and the protein levels of TNF-α, IL-6, and MCP-1 in renal tissues were substantially increased by LPS stimulation, while treatment with MaR1 reduced their expression correspondingly. These results indicated that MaR1 could mitigate renal inflammation efficaciously in LPS-induced S-AKI mice.

### MaR1 Attenuates Cell Apoptosis in Kidneys of LPS-Induced S-AKI Mice

Renal cell apoptosis is another protruding feature in the pathogenesis of S-AKI. To investigate the effect of MaR1 on kidney apoptosis, TUNEL staining was employed. As exhibited in Figures 2E,F, apoptotic cell nuclei were observed in kidney slices of the LPS group, and MaR1 administration diminished the number of TUNEL-positive cells, indicating that MaR1 could inhibit renal cell apoptosis in LPS-related AKI mice. In the meantime, the expression of cleaved caspase-3, a key executioner which modified proteins responsible for apoptosis, was remarkably reduced by MaR1 treatment by immunofluorescence staining and Western blotting assay (Figures 2E,H,I). Correspondingly, MaR1 downregulated the mRNA and protein ratios of BAX/BCL-2 (Figures 2G,I), downregulated BAX protein, and upregulated BCL-2 protein compared to those of LPS group (Figures 2H,I). In summary, these outcomes revealed that MaR1 played an anti-apoptotic role in the kidneys of LPS-stimulated septic AKI mice.

### MaR1 Ameliorates LPS-Induced Renal Oxidative Stress Injury in S-AKI Mice

**FIGURE 3:**
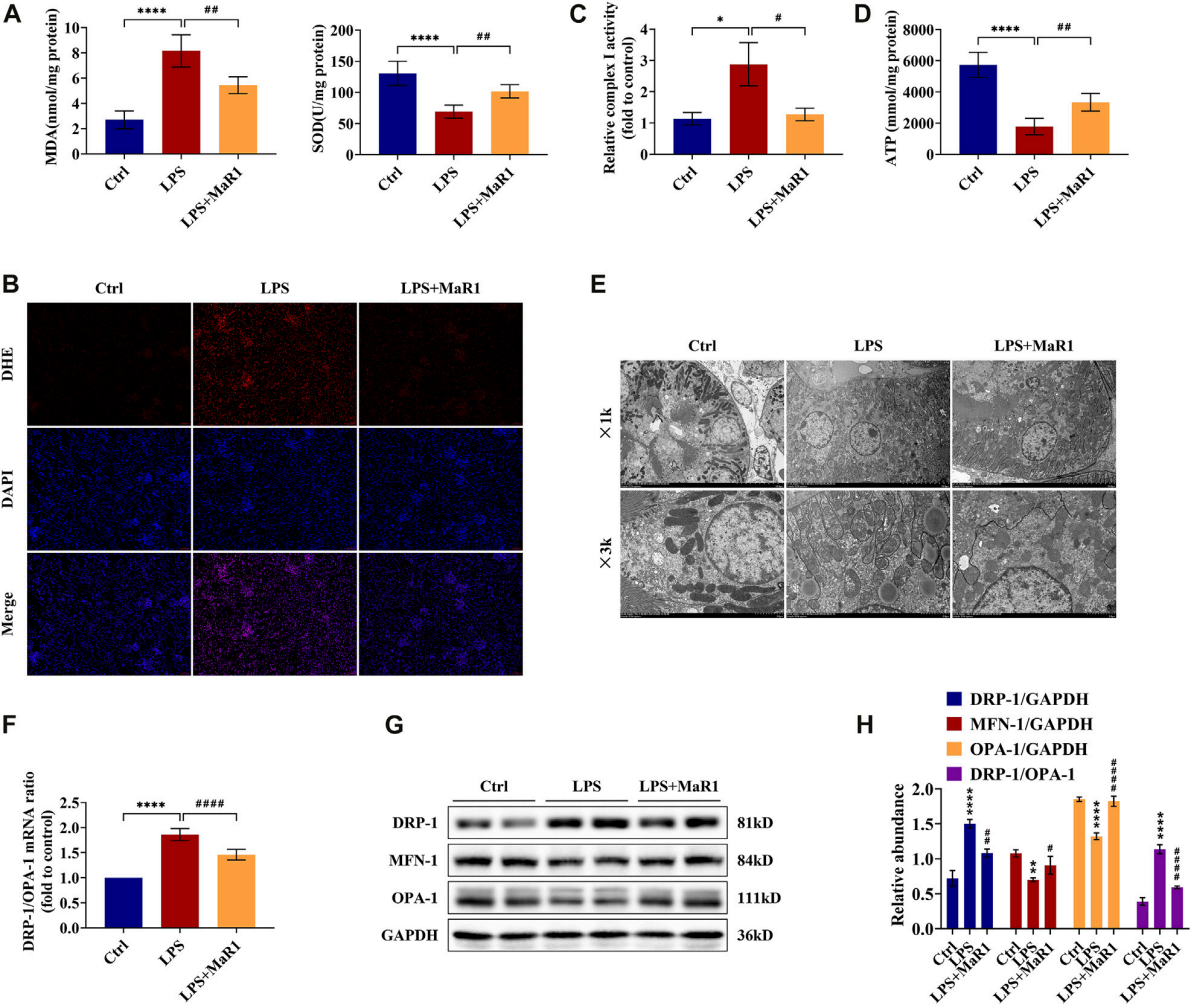
MaR1 attenuated oxidative stress and protected mitochondrial quality in the kidneys of lipopolysaccharide (LPS)-induced S-AKI mice. **(A)** The levels of malondialdehyde and superoxide dismutase of renal tissues in each group of mice were detected using assay kits. **(B)** Reactive oxygen species were assessed *in situ* by staining (scale bar = 50 μm). **(C)** The relative complex I activity of renal tissues in each group of mice was evaluated using assay kits. **(D)** The ATP production of renal tissues was detected using assay kits. **(E)** Photomicrographs were collected by transmission electron microscopy (1k and 3k). **(F)** The gene expression ratio of DRP-1/OPA-1 in renal tissues was quantitated using qRT-PCR. **(G)** The expressions of DRP-1, MFN-1, and OPA-1 in renal tissues were analyzed using Western blot. **(H)** The protein levels of DRP-1, MFN-1, and OPA-1 were quantified by densitometry and normalized with GAPDH (time point: 12 h). Data are presented as mean ± SD, *n* = 5. *p < 0.05, **p < 0.01, ***p < 0.001, ****p < 0.0001 vs Control group; #*p* < 0.05, ##*p* < 0.01, ###*p* < 0.001, ####*p* < 0.0001 vs LPS group.

Oxidative stress is an important pathogenic mechanism in the development of septic AKI. As shown in Figure 3A, the level of MDA in kidney tissue was induced by LPS administration, while MaR1 significantly reduced the increase of MDA. At the same time, the SOD level of the LPS group diminished remarkably, while treatment with MaR1 effectively alleviated the decrease in SOD concentration. As demonstrated in Figure 3B, the levels of ROS (indicated by red fluorescence) were dramatically higher in the renal tissue of the LPS group than that of the control group, and a profound decrease was observed in the MaR1 group. The activity of the mitochondrial complex I could reflect ROS production and oxidative damage. In the LPS group, the mitochondrial complex I activity was activated to nearly threefolds compared to that of the control (*p* < 0.05), while MaR1 treatment could suppress this activation effectively (*p* < 0.05) (Figure 3C). These results together suggested that MaR1 could effectively inhibit LPS-induced oxidative stress injury in S-AKI mice.

### MaR1 Improves Mitochondrial Function, Structure, and Dynamics Balance of LPS-Induced S-AKI Mice

To explore the protective mechanism of MaR1 on LPS-induced AKI, we focused on mitochondrial function and dynamics balance. As shown in Figure 3D, we assessed the ATP production ability of kidney tissue and found the ATP production of LPS mice to be remarkably decreased compared with that of the control mice, while MaR1 restored the ATP production ability of the mitochondria (*p* < 0.01). Additionally, a transmission electron microscopy observation was performed, which showed that the mitochondria of LPS-treated mice in kidney tubular cells exhibited a more severely injured morphology, such as more mitochondrial fragmentation, loss of cristae, fragmentation, swelling, and vacuoles in the mitochondrial matrix, compared with those of the normal control group. These injuries were alleviated by MaR1 administration remarkably (Figure 3E). Moreover, to demonstrate the change of mitochondrial dynamics balance in different groups, we detected the expressions of dynamic regulatory-related mRNAs and proteins. Mitochondrial fission protein DRP-1 and mitochondrial fusion proteins MFN-1 and OPA-1 were detected by qRT-PCR and Western blot. The results showed that the protein level of DRP-1 and the mRNA and protein ratios of DRP-1/OPA-1 were upregulated, and MFN-1 and OPA-1 were downregulated following LPS treatment, while MaR1 could reverse these trends (Figures 3F–H). All these data suggested that MaR1 could achieve a renoprotective effect through regulating mitochondrial function, structure, and dynamics balance.

### MaR1 Reduces Kidney Damage by Inhibiting the Expression of NOX4 and the NF-κB p65 Signaling Pathway of S-AKI Mice

**FIGURE 4:**
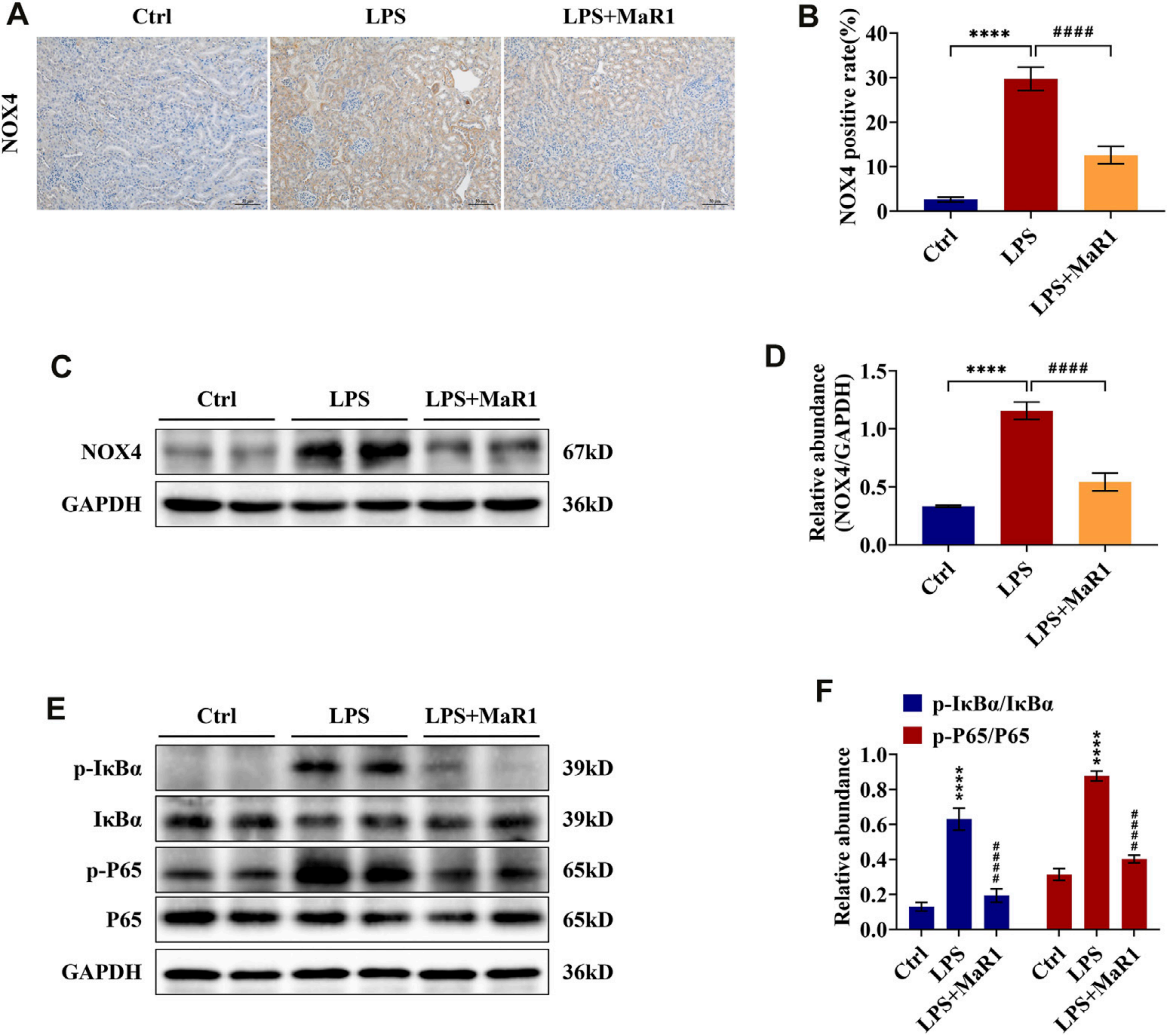
MaR1 inhibited NOX4 expression and NF-κB pathway activation in the kidneys of lipopolysaccharide (LPS)-induced S-AKI mice. **(A)** Representative immunohistochemistry images of NOX4 in renal sections were collected (scale bar = 50 μm). **(B)** Average optical density of NOX4. **(C)** The expression of NOX4 in renal tissues was analyzed using Western blot. **(D)** The protein level of NOX4 was quantified by densitometry and normalized with GAPDH. **(E)** The expressions of p-IκBα, IκBα, p-P65, and P65 in renal tissues were analyzed using Western blot. **(F)** The protein levels of p-IκBα/IκBα and p-P65/P65 were quantified by densitometry and normalized with GAPDH (time point: 12 h). Data are presented as mean ± SD, *n* = 5. *p < 0.05, **p < 0.01, ***p < 0.001, ****p < 0.0001 *vs*. control group; #*p* < 0.05, ##*p* < 0.01, ###*p* < 0.001, ####*p* < 0.0001 *vs*. LPS group.

To identify the anti-inflammatory, anti-apoptotic, and anti-oxidative stress mechanism of MaR1 in the kidneys of LPS-induced S-AKI mice, the activation of the signaling pathways was further explored by immunohistochemistry and Western blotting analysis. We found that the protein expression of NOX4 was significantly upregulated, as shown in Figures 4A–D, whereas it was markedly suppressed by MaR1 treatment (*p* < 0.0001). Besides this, the phosphorylation levels of IκBα and NF-κB p65 in the kidneys of the MaR1 group were obviously lower than those of the LPS group (both *p* <0.0001) (Figures 4E,F), which suggested that MaR1 could inhibit the activated NF-κB p65 pathways. These findings implied that MaR1 played anti-inflammatory, anti-apoptotic, and anti-oxidative stress roles by inhibiting NOX4 expression and the activation of the NF-κB p65 pathway in the kidneys of LPS-stimulated S-AKI mice.

### MaR1 Promotes Cell Survival in LPS-Stimulated TCMK-1 Cells

**FIGURE 5:**
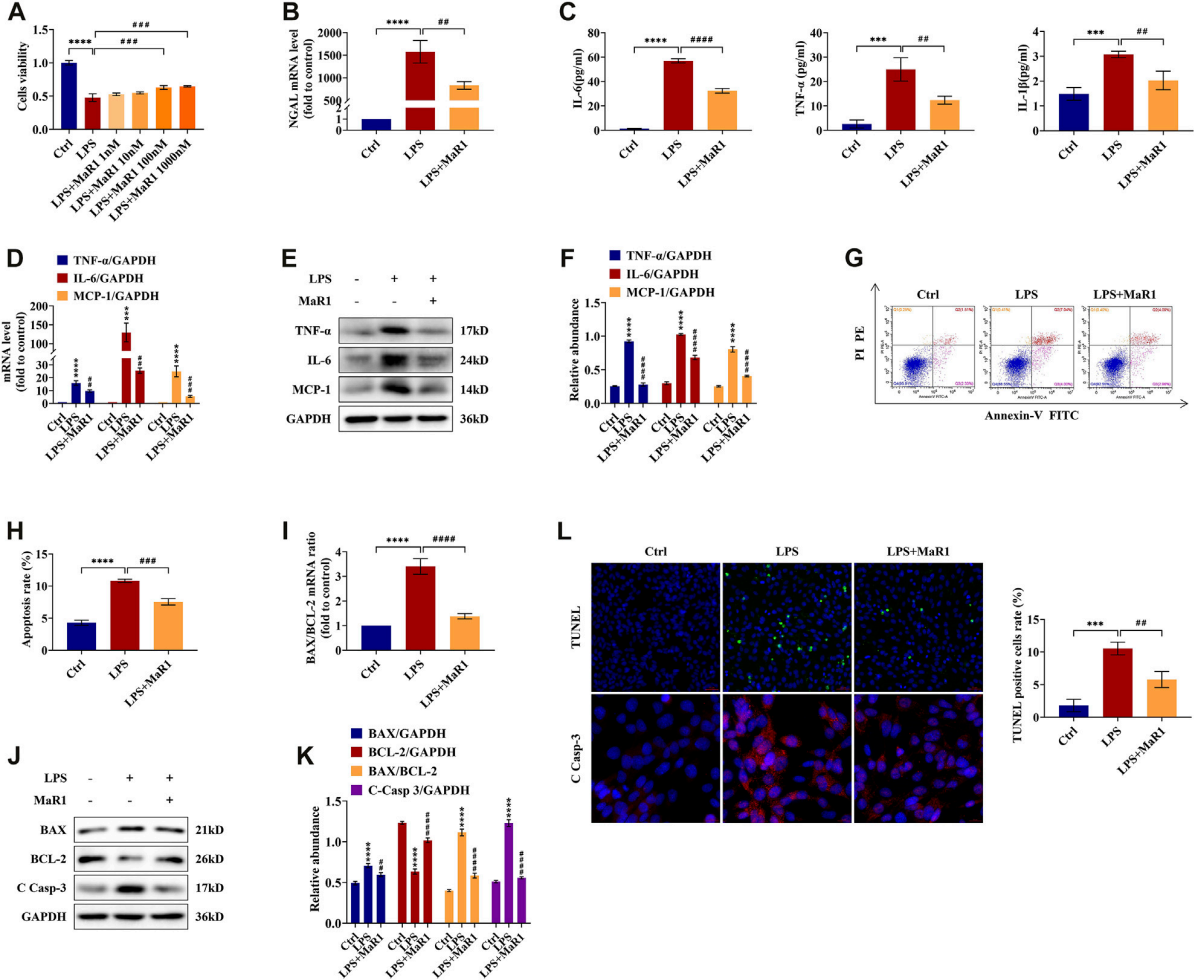
MaR1 promoted cell survival and prevented the inflammation and apoptosis of lipopolysaccharide (LPS)-stimulated TCMK-1 cells. **(A)** The effects of LPS (100 μg/ml) and MaR1 (1, 10, 100, and 1,000 nM) on TCMK-1 cells were determined using CCK-8. **(B)** The gene expression of NGAL in TCMK-1 cells was quantitated using qRT-PCR. **(C)** The cell supernatant levels of IL-6 and TNF-α, and IL-1β were determined using assay kits in each group of TCMK-1 cells. **(D)** The gene expressions of TNF-α, IL-6, and MCP-1 in TCMK-1 cells were quantitated using qRT-PCR. **(E)** The expressions of TNF-α, IL-6, and MCP-1 in TCMK-1 cells were analyzed using Western blot. **(F)** The protein levels of TNF-α, IL-6, and MCP-1 were normalized with GAPDH. **(G)** Flow cytometry analysis of apoptosis using Annexin V-FITC and PI staining. **(H)** Percentage of apoptosis in each group of TCMK-1 cells. **(I)** The gene expression ratio of BAX/BCL-2 in TCMK-1 cells was quantitated using qRT-PCR. **(J)** The expressions of BAX, BCL-2, and C Casp-3 in TCMK-1 cells were analyzed using Western blot. **(K)** The protein levels of BAX, BCL-2, and C Casp-3 were normalized with GAPDH. **(L)** TUNEL staining (scale bar = 50 μm), percentage of TUNEL-positive cells (%), and immunofluorescence staining of C Casp-3 expression (scale bar = 20 μm) of TCMK-1 cells (time point: 24 h). Data are presented as mean ± SD, *n* = 5. *p < 0.05, **p < 0.01, ***p < 0.001, ****p < 0.0001 *vs*. control group; #*p* < 0.05, ##*p* < 0.01, ###*p* < 0.001, ####*p* < 0.0001 *vs*. LPS group.

We further investigated the anti-inflammatory and anti-apoptotic role of MaR1 *in vitro*. To explore the optimum concentration of MaR1 administration in TCMK-1 cells exposed to LPS, MaR1 concentrations of 1, 10, 100, and 1,000 nM were determined by the CCK-8 assay. As shown in Figure 5A, the cell viability in the LPS groups was significantly lower than that in normal control (*p* < 0.0001). With 100 nM MaR1 pretreatment for 30 min, the cell viability was significantly improved compared to that in the LPS group (*p* < 0.001). When MaR1 concentration exceeded 100 nM, there was no further amelioration. Hence, we employed 100 nM as the MaR1 administration concentration in subsequent experiments. Furthermore, the qRT-PCR result demonstrated that pretreatment with MaR1 obviously reduced the increased NGAL mRNA level of TCMK-1 cells in the LPS group (*p* < 0.01) (Figure 5B), which was consistent with our *in vivo* findings.

### MaR1 Prevents Inflammation and Apoptosis in LPS-Stimulated TCMK-1 Cells

TCMK-1 cells and supernatant were collected for detecting the expressions of TNF-α, IL-6, IL-1β, and MCP-1. As shown in Figure 5C, the supernatant levels of TNF-α, IL-6, and IL-1β in the MaR1 group were evidently lower than those in the LPS group. The mRNA and protein levels of TNF-α, IL-6, and MCP-1 were likewise upregulated in the LPS group and reversed in the MaR1 group (Figures 5D–F). Flow cytometry and TUNEL staining were employed to explore the anti-apoptotic role of MaR1 in LPS-induced TCMK-1 cells, and we found that MaR1 pretreatment diminished the increased apoptosis rate and TUNEL-positive cells of the LPS group (Figures 5G,H,L). Besides these, MaR1 could decrease the mRNA and protein ratios of BAX/BCL-2 (Figures 5I,K), downregulated BAX protein, and upregulated BCL-2 protein compared to those of the LPS group (Figures 5J,K). In addition, the expression of cleaved caspase-3 was significantly reduced by MaR1 administration by immunofluorescence staining and Western blotting assay (Figures 5J–L). Taken together, these results confirmed that MaR1 inhibited the development of inflammation and apoptosis as well as recovered the cell activity in LPS-stimulated TCMK-1 cells.

### MaR1 Reduces Mitochondrial Membrane Potential Loss and ROS Level in LPS-Stimulated TCMK-1 Cells

**FIGURE 6:**
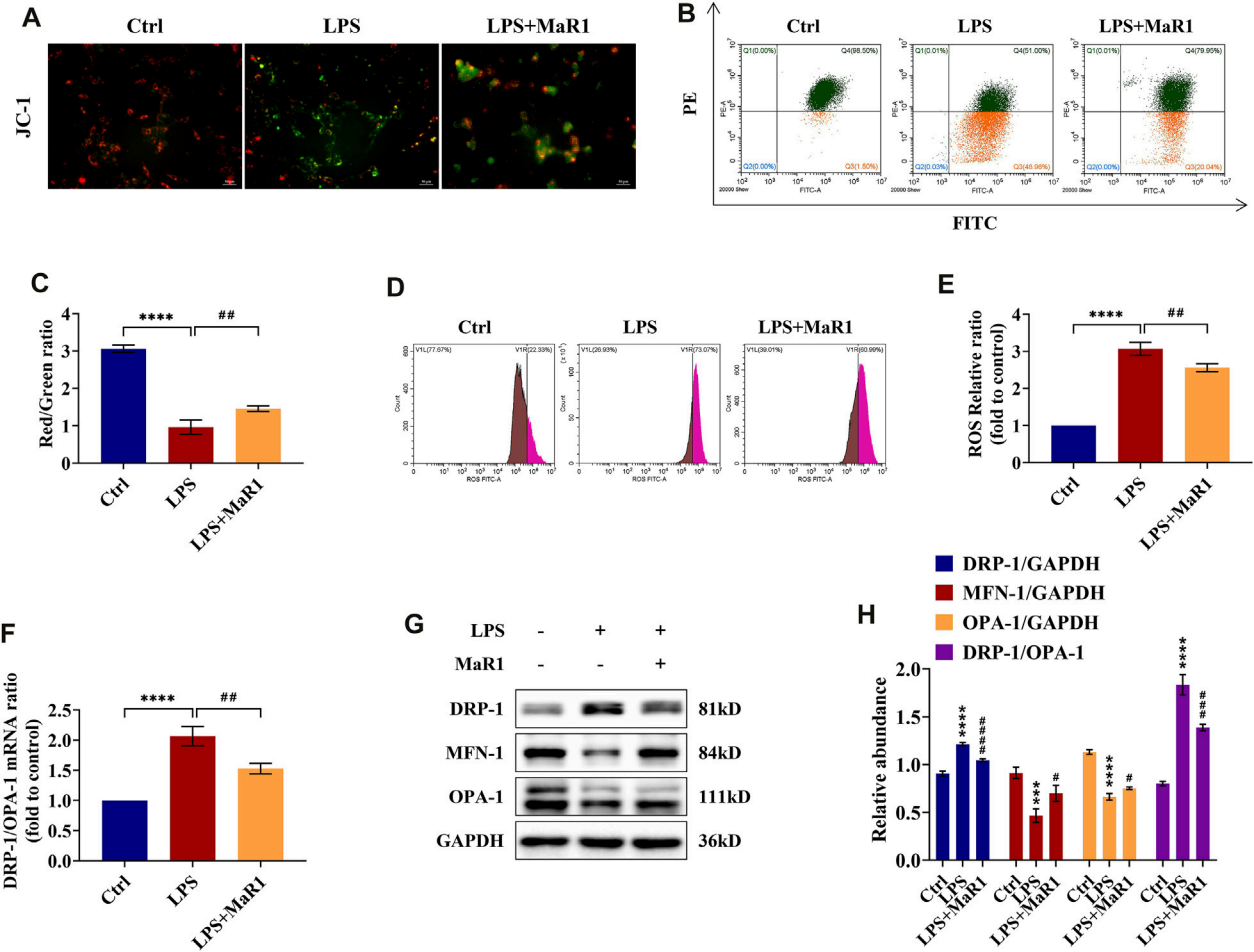
MaR1 preserved the mitochondrial quality and reduced the reactive oxygen species (ROS) levels of lipopolysaccharide (LPS)-stimulated TCMK-1 cells. **(A)** Representative pictures of JC-I staining of TCMK-1 cells (scale bar = 50 μm). **(B)** Flow cytometry analysis of MMP using JC-1 staining. **(C)** Red/green fluorescence ratio of JC-1 staining of TCMK-1 cells in each group. **(D)** The ROS levels of TCMK-1 cells were determined by flow cytometry. **(E)** ROS relative ratio of TCMK-1 cells in each group. **(F)** The gene expression ratio of DRP-1/OPA-1 in TCMK-1 cells was quantitated using qRT-PCR. **(G)** The expressions of DRP-1, MFN-1, and OPA-1 in TCMK-1 cells were analyzed using Western blot. **(H)** The protein levels of DRP-1, MFN-1, and OPA-1 were quantified by densitometry and normalized with GAPDH (time point: 24 h). Data are presented as mean ± SD, *n* = 5. *p < 0.05, **p < 0.01, ***p < 0.001, ****p < 0.0001 *vs*. control group; #*p* < 0.05, ##*p* < 0.01, ###*p* < 0.001, ####*p* < 0.0001 *vs*. LPS group.

The decline of MMP is a sign for the early stage of apoptosis. We used fluorescence microscopy and flow cytometry to evaluate the effects of MaR1 on the MMP of TCMK-1 cells in LPS-induced injury. The results showed that the MMP of TCMK-1 cells subjected to LPS displayed a decline compared to the control group, while it could be partially reversed by MaR1 pretreatment (*p* < 0.0001) (Figures 6A–C). ROS are specific drivers of mechanism contributing to mitochondria damage. As measured by flow cytometry, we found that the ROS levels were significantly increased in the LPS group, while they could be decreased *via* administration of MaR1 (*p* < 0.01) (Figures 6D,E).

### MaR1 Preserves the Mitochondrial Dynamics Balance in LPS-Stimulated TCMK-1 Cells

Corresponding to the experiments *in vivo*, we assessed the mRNA and protein expressions of DRP-1, MFN-1, and OPA-1 in TCMK-1 cells by qRT-PCR and Western blot. The data indicated that MaR1 could reverse the changes of DRP-1, MFN-1, and OPA-1 protein levels as well as the mRNA and protein ratios of DRP-1/OPA-1 towards those of the normal control group (Figures 6F–H), which were corroborated with *in vivo* findings. Thus, the mitochondrial homeostasis of LPS-stimulated TCMK-1 cells could be maintained by pretreatment of MaR1.

### MaR1 Abrogated the Activation of NOX4 and NF-κB p65 Signaling Pathway in LPS-Stimulated TCMK-1 Cells

**FIGURE 7:**
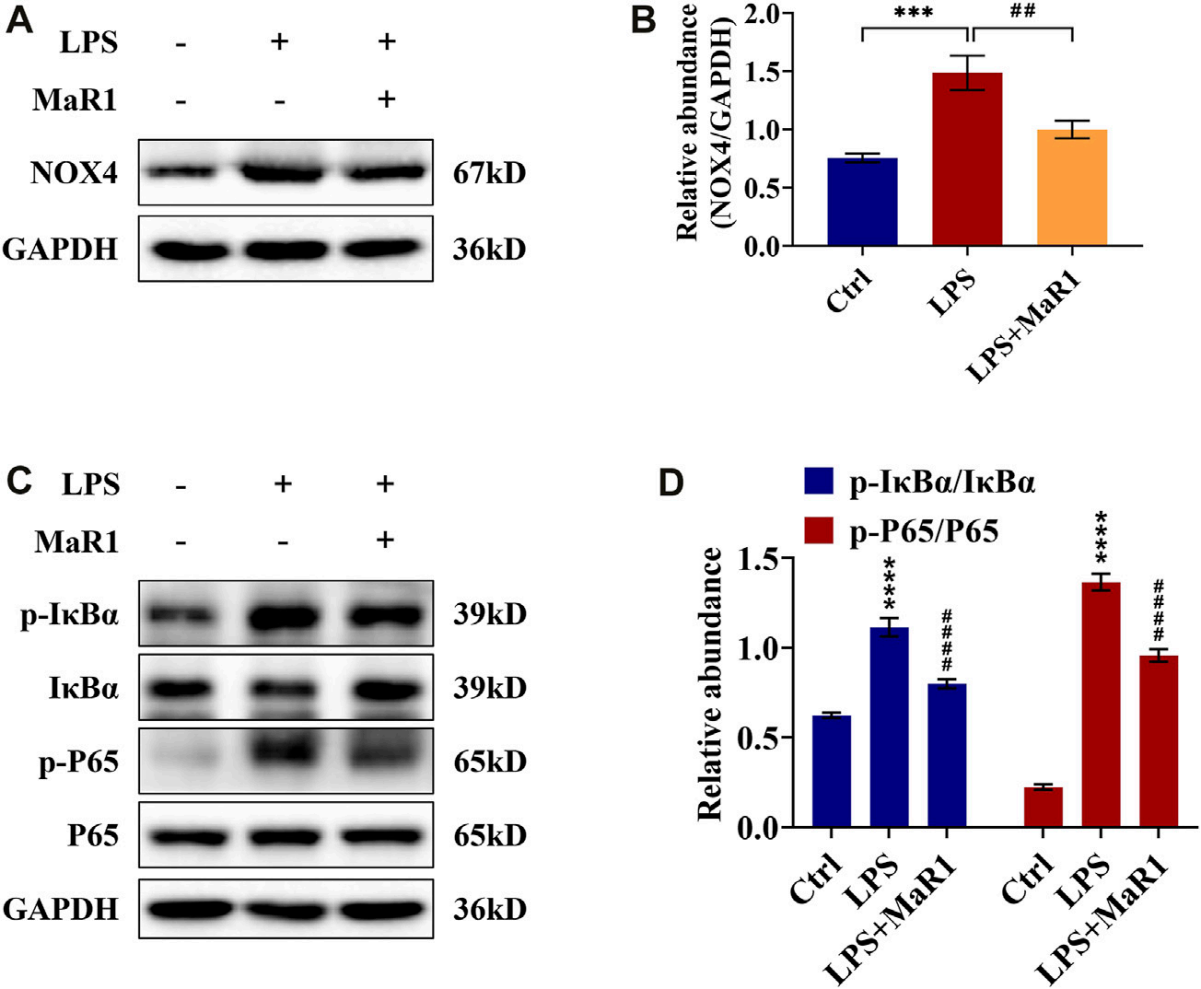
MaR1 suppressed NOX4 expression and NF-κB pathway activation of lipopolysaccharide (LPS)-stimulated TCMK-1 cells. **(A)** The expression of NOX4 in TCMK-1 cells was analyzed using Western blot. **(B)** The protein level of NOX4 in TCMK-1 cells was quantified by densitometry and normalized with GAPDH. **(C)** The expressions of p-IκBα, IκBα, p-P65, and P65 in TCMK-1 cells were analyzed using Western blot. **(D)** The protein levels of p-IκBα/IκBα and p-P65/P65 were quantified by densitometry and normalized with GAPDH (time point: 24 h). Data are presented as mean ± SD, *n* = 5. *p < 0.05, **p < 0.01, ***p < 0.001, ****p < 0.0001 *vs*. control group; #*p* < 0.05, ##*p* < 0.01, ###*p* < 0.001, ####*p* < 0.0001 *vs*. LPS group.

To determine the potential signaling mechanism of MaR1 in LPS-stimulated TCMK-1 cells, we also assessed the expression of the NOX4 and NF-κB p65 signaling pathway in TCMK-1 cells. As shown in Figures 7A,B, Western blotting analysis confirmed that the upregulated protein expression of NOX4 in the LPS group was significantly suppressed by MaR1 pretreatment (*p* < 0.01). Moreover, the phosphorylation levels of IκBα and NF-κB p65 in the TCMK-1 cells of the MaR1 group were remarkably lower than those of the LPS group (both *p* < 0.0001) (Figures 7C,D). These data demonstrated that MaR1 exhibited a certain protective effect on TCMK-1 cells by suppressing the activation of NOX4 and NF-κB p65 signaling pathway.

## Discussion

Sepsis is the most common cause of AKI in critically ill patients, and S-AKI is associated with unacceptable in-hospital mortality (Uchino et al., 2005; Hoste et al., 2015). However, the mechanism by which sepsis induces AKI is poorly understood, and many therapeutic interventions have been unsatisfactory. Inflammation always accompanies infection during sepsis. The inflammatory response inherent to sepsis must be considered as a direct mechanism of AKI (Wang et al., 2012; Quoilin et al., 2014; Maiden et al., 2016). Hence, the research and development of anti-inflammatory drugs have become attractive in recent years. Recent studies have already confirmed the role of pro-resolving mediators derived from polyunsaturated fatty acids in modulating inflammatory reaction (Serhan, 2014; Leuti et al., 2019). MaR1, a newly discovered endogenous lipid mediator, has been reported to play an important role in inflammation regression, tissue homeostasis, wound healing, and host defense (Gong et al., 2014; Colas et al., 2016; Francos-Quijorna et al., 2017; Laiglesia et al., 2018). In this study, we observed the protective effects of MaR1 against LPS-induced S-AKI in preserving renal function and alleviating pathological injury, which provided a new therapeutic approach for septic AKI.

Inflammation is the primary defense mechanism of a host from invading pathogens. However, an imbalanced inflammatory reaction may lead to organ failure and poor outcome (Peerapornratana et al., 2019). It has been proved that the harmful inflammatory cascade of sepsis plays a pivotal role in the occurrence and development of S-AKI (Hotchkiss and Karl, 2003; Zarjou and Agarwal, 2011). In the immune response to sepsis, exogenous factors derived from the pathogen (*e*.*g*., LPS) and endogenous factors released by injured cells (*e*.*g*., high-mobility group box-1 protein) can interact with various pattern recognition receptors such as Toll-like receptors (TLRs) and C-type lectin receptors, then upregulate the expression of inflammation-related genes, and trigger the production of inflammatory cytokines, such as IL-1, IL-6, TNF-α, and adaptor protein 1 (Kawai and Akira, 2010; Lamkanfi, 2011; Raymond et al., 2017). Up to now, plenty of research have demonstrated that anti-inflammatory agents could be of advantage to S-AKI (Duffield et al., 2006; Zhao et al., 2016). In the current study, MaR1 treatment significantly restricted inflammation *via* downregulating the expressions of TNF-α, IL-6, IL-1β, and MCP-1 in LPS-stimulated AKI kidneys and TCMK-1 cells. These results are consistent with other studies. Furthermore, inflammatory infiltration would induce the occurrence of cellular apoptosis, which caused the loss of renal epithelial cells that characterized AKI (Havasi and Borkan, 2011). Here we detected the levels of BAX, BCL-2, and cleaved caspase-3, and the data showed that BAX and cleaved caspase-3 expressions were suppressed, while BCL-2 was preserved, in the S-AKI kidneys and TCMK-1 cells after pretreatment with MaR1. TUNEL staining and flow cytometry assays also indicated that MaR1 evidently alleviated the apoptosis of tubular epithelial cells compared with LPS administration alone. Therefore, MaR1 could prevent inflammation and apoptosis effectively in LPS-induced S-AKI model.

The mitochondria play key roles in the production of ATP and preservation of redox homeostasis. The mitochondria are also a main intracellular source and a primary target of ROS, which destroys the mitochondrial membrane and makes them extremely vulnerable to injury under stressful conditions. Various quality control mechanisms have evolved in the mitochondria to resist stress and maintain integrity and function, such as mitochondrial dynamics (fusion and fission), mitophagy, and protein quality control (Tang et al., 2021). Loss of mitochondrial quality control may induce mitochondrial damage and dysfunction, leading to cell death, tissue injury, and possible organ failure (Suliman and Piantadosi, 2016; Bhargava and Schnellmann, 2017). Accumulating evidence implies that mitochondrial dysfunction makes a crucial contribution to the pathogenesis of AKI (Mukhopadhyay et al., 2012; Dare et al., 2015). Moreover, mitochondrial fragmentation resulting from excessive fission and/or suppression of fusion has been implicated as a key event in mitochondrial damage and kidney tubule injury during AKI (Brooks et al., 2009; Wei et al., 2018). The experimental results in our study indicated that LPS administration damaged the mitochondria quality in kidney and TCMK-1 cells, which were characterized by increasing ROS levels, decreasing ATP level, and decline of MMP as well as fission activation and fusion suppression. After MaR1 treatment, the above-mentioned changes were reversed to some extent. Taken together, these findings demonstrated that MaR1 is capable to preserve healthy mitochondrial quality.

In the present study, we firstly found that the increased NOX4 in S-AKI mice and TCMK-1 cells could be significantly downregulated after the intervention of MaR1. NOX4 is expressed mainly in proximal tubular cells with lower levels in the glomerulus (Geiszt et al., 2000). NADPH oxidase catalyzes the transfer of electrons from NADPH to molecular oxygen through the NOX catalytic subunit to produce ROS like O_2_^−^ and H_2_O_2_, which is the primary function of NOX4 (Yoo et al., 2020). NADPH oxidase-derived ROS could function as secondary messengers to activate the NF-κB signaling transduction pathway (Li et al., 2014; Zhu et al., 2021). The NF-κB p65 pathway is a central contributor of inflammation response in the kidneys. Nuclear translocation and transcriptional activation of NF-κB are mainly dependent on IκBα phosphorylation. This study displayed that the ROS levels and expressions of phosphorylation of IκBα and p65 in the MaR1 group were significantly reduced compared with the LPS group, which indicated that MaR1 could inhibit the production of ROS and the activation of the NF-κB p65 pathway. In summary, we found that MaR1 may play a vital role in anti-inflammation, anti-oxidative stress, and renal protection *via* the NOX4/ROS/NF-κB signaling pathway. In addition, a 2019 study demonstrated that MaR1 is renoprotective in a mouse model of ischemia–reperfusion injury, attenuating inflammation and oxidative stress by inhibiting the TLR4/MAPK/NF-κB pathway and activating the Nrf2 pathway (Qiu et al., 2019). *In vitro* studies from a 2017 article demonstrated the ability of MaR1 to inhibit high-glucose-induced fibrotic responses in mesangial cells, suggesting that MaR1 may also function as an antifibrotic molecule (Tang et al., 2017). These data indicate that MaR1 has additional cellular sites of action in the kidney that contribute to their pro-resolving activity.

Maresins (macrophage mediators in resolving inflammation) are derived from the omega-3 fatty acid DHA (Serhan et al., 2009). MaRs are produced by macrophages *via* initial lipoxygenation at the carbon-14 position by the insertion of molecular oxygen, producing a 13S,14S-epoxide–maresin intermediate that is enzymatically converted to maresin family members maresin1, maresin 2, and maresin conjugate in tissue regeneration (Serhan, 2017; Serhan et al., 2018). In addition to counter-regulating the proinflammatory cytokines and blocking the NF-κB gene products, MaRs could limit the further recruitment of PMNs and inhibit neutrophil infiltration *in vivo* yet stimulate the nonphlogistic recruitment of mononuclear cells. When macrophages encounter MaRs, they increase phagocytosis and efferocytosis, resulting in the removal of microbes, and they clear PMNs from the sites (Serhan, 2017). Furthermore, SPMs have already been administered in human trials and indicated exciting effects. A double-blinded, placebo-controlled, crossover study in 2020 demonstrated that supplementation with refined marine oils could lead to a rapid upregulation of peripheral blood SPM concentrations and reprograming of peripheral blood cell responses to sterile and infectious stimuli. These changes were found to persist after the SPM concentrations returned back to baseline. This study also established a correlation between SPMs and the regulation of platelet, monocyte, and neutrophil responses, thereby providing potential novel biomarkers for establishing the efficacy of marine oil supplementation in controlling host immune responses (Souza et al., 2020). We are expecting more basic research and clinical research on MaRs and other SPMs to provide new approaches to treating inflammation-associated diseases.

Besides the above-mentioned findings, there are certain limits in the study. First, we established a septic mouse model only by one shot injection of LPS, which is different from recurrent pulse endotoxin release in patients with sepsis. A model of cecal ligation and puncture would also be needed in our future research to investigate whether MaR1 treatment could affect the removal of microbes. Moreover, we only employed TCMK-1 cells, while other renal parenchymal cells such as glomerular endothelial cells or podocytes were not observed *in vitro*. Therefore, more research is needed to fully clarify the specific protection mechanism of MaR1 for S-AKI.

## Conclusion

Our results confirmed that MaR1 is capable to alleviate LPS-induced S-AKI by reducing pro-inflammatory cytokines and cell apoptosis, together with the preservation of mitochondrial quality. This might be related to the inhibition of the NOX4/ROS/NF-κB pathways. MaR1 may be proposed as one of the potential therapeutic agents to treat septic AKI.

## Data Availability

The raw data supporting the conclusions of this article will be made available by the authors, without undue reservation.

## Abbreviations

S-AKI, sepsis-associated acute kidney injury; LPS, lipopolysaccharide; MaR1, maresin 1; Scr, serum creatinine; BUN, blood urea nitrogen; HE, hematoxylin and eosin; NGAL, neutrophil gelatinase-associated lipocalin; TNF-α, tumor necrosis factor-α; IL, interleukin; ROS, reactive oxygen species; MCP-1, monocyte chemoattractant protein-1; GAPDH, glyceraldehyde phosphate dehydrogenase; BCL-2, B-cell lymphoma-2; BAX, BCL2-associated X; C Casp-3, cleaved capase-3; MDA, malondialdehyde; SOD, superoxide dismutase; ATP, adenosine triphosphate; DRP-1, dynamin-related protein-1; OPA-1, optic atrophy-1; MFN-1, mitofusin1; NADPH, nicotinamide adenine dinucleotide phosphate; NOX4, NADPH oxidase 4; IκBα, inhibitor kappa B alpha; NF-κB, nuclear factor-kappa B; IHC, immunohistochemistry; IF, immunofluorescence; CCK-8, cell counting kit 8; DAPI, 4,6-diamidino-2-phenylindole
